# Cardiac Progenitor Cells and the Interplay with Their Microenvironment

**DOI:** 10.1155/2017/7471582

**Published:** 2017-09-17

**Authors:** Arianna Mauretti, Sergio Spaans, Noortje A. M. Bax, Cecilia Sahlgren, Carlijn V. C. Bouten

**Affiliations:** ^1^Department of Biomedical Engineering, Eindhoven University of Technology, P.O. Box 513, 5600 MB Eindhoven, Netherlands; ^2^Institute for Complex Molecular Systems, Eindhoven University of Technology, Eindhoven, Netherlands; ^3^Faculty of Science and Engineering, Åbo Akademi University, 20520 Turku, Finland; ^4^Turku Centre for Biotechnology, Åbo Akademi University and University of Turku, 20520 Turku, Finland

## Abstract

The microenvironment plays a crucial role in the behavior of stem and progenitor cells. In the heart, cardiac progenitor cells (CPCs) reside in specific niches, characterized by key components that are altered in response to a myocardial infarction. To date, there is a lack of knowledge on these niches and on the CPC interplay with the niche components. Insight into these complex interactions and into the influence of microenvironmental factors on CPCs can be used to promote the regenerative potential of these cells. In this review, we discuss cardiac resident progenitor cells and their regenerative potential and provide an overview of the interactions of CPCs with the key elements of their niche. We focus on the interaction between CPCs and supporting cells, extracellular matrix, mechanical stimuli, and soluble factors. Finally, we describe novel approaches to modulate the CPC niche that can represent the next step in recreating an optimal CPC microenvironment and thereby improve their regeneration capacity.

## 1. Introduction

**Figure 1:**
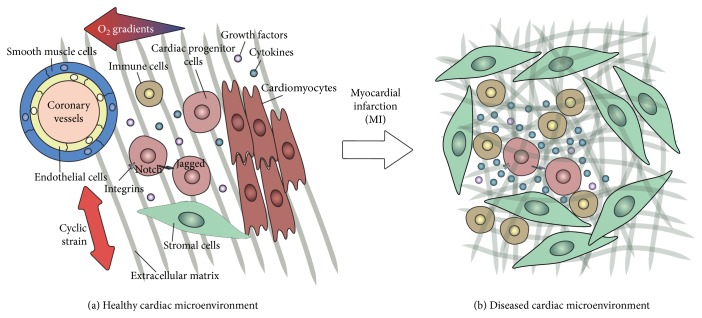
The cardiac progenitor cell resident microenvironment. (a) The simplified representation shows some of the key players of the healthy CPC niche: (1) cellular elements (CPCs and supporting cells: cardiomyocytes, endothelial cells, smooth muscle cells, stromal cells, and immune cells) and cell-cell interactions such as signaling via Notch; (2) extracellular matrix (ECM); (3) mechanical stimuli, such as the cyclic strain provided by the beating heart; and (4) soluble factors, such as cytokines, oxygen gradients, and growth factors. (b) Simplified representation of the infarcted heart, where the microenvironment is altered and the niche components modified: (1) cardiomyocyte death and infiltration of myofibroblasts and immune cells; (2) excessive and disordered formation of ECM; (3) increased ECM stiffness and thus altered mechanical behavior; and (4) increased secretion of growth factors and cytokines.

Cardiac tissue is a composite material consisting of contractile and supportive cells surrounded by extracellular matrix (ECM) and is intertwined with nervous and vascular networks. An ischemic event, such as a myocardial infarction (MI), not only induces cell death but also affects the tissue structure and composition. This can eventually lead to loss of cardiac function due to changes in the key players of the cardiac microenvironment: (1) stem/progenitor cells and supporting cells, (2) extracellular matrix (ECM) proteins, (3) the mechanical environment of the cells and the matrix, such as the cyclic strain provided by the beating heart, and (4) soluble factors, such as oxygen and cytokines (Figure 1(a)). In this review, we omit to describe vascular components, innervation, and electrical conduction, as these are extensively reviewed elsewhere [1–3], although their derivatives, such as oxygen gradients and cyclic strain, are included.

The myocardium shows very limited self-renewal; nevertheless, the notion of the heart as a terminally differentiated organ, incapable of regenerating after injury, has been challenged by abundant evidence in the last decade [4, 5]. There is ongoing debate over whether cardiac regeneration is to be attributed to dedifferentiation and proliferation of cardiomyocytes [6, 7] or to differentiation of cardiac stem or progenitor cells [8–10], which makes it difficult to identify the ideal therapeutic target. Nevertheless, the existence of resident cardiac progenitor cells (CPCs) in the heart and their relevance for cardiac regeneration have been demonstrated by several studies [10–13]. CPCs have emerged as a promising candidate for cardiac regeneration, due to their differentiation potential [10, 14] and the ability to produce and remodel ECM proteins [15]. Moreover, after acute MI, the number of CPCs in the adult increases and differentiation into the cardiac lineages takes place [6]. However, the post-MI microenvironment can affect CPC behavior: in chronic infarcts, CPCs are characterized by decreased telomerase activity, leading to impaired cell division and cellular senescence, as well as increased CPC apoptosis [6].

The traditional cell therapy approach to treat a MI entails isolation of CPCs, their expansion *in vitro*, and transplantation into the infarcted area [16]. Despite the immediate benefits on cardiac function, this treatment has shown very limited improvement on the long term [17–19], mainly due to low cell survival and engraftment in the host tissue [20]. In fact, a MI creates a hostile environment for the injected progenitor cells, due to the inflammatory response and tissue alterations, such as scar tissue formation, triggered by the cardiac injury, as extensively described elsewhere [20–22] (Figure 1(b)).

In the adult, stem or progenitor cells reside in specific microenvironments, referred to as “niches,” that protect stem cells and regulate their fate and functions [23–25]. Stem cell niches are stored in specific anatomical compartments, located in tissue areas that are shielded from external damaging stimuli [23, 26].

In the adult mouse heart, putative progenitor cell niches have been identified in the atria, base-mid region, and apex [27]. To date, however, the cardiac progenitor cell niche is still largely uncharacterized and most studies have been performed on mouse models [27, 28].

**Figure 2:**
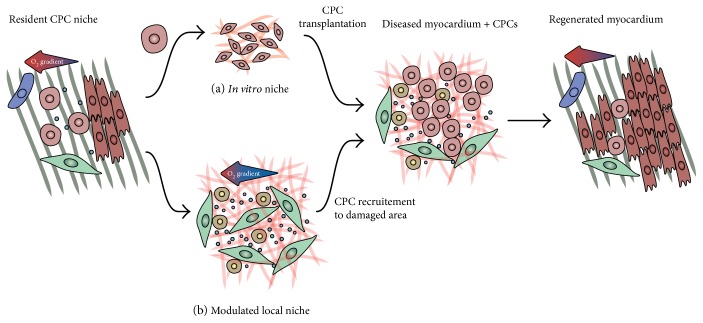
The CPC microenvironments. For therapeutic application, CPCs can be isolated from their resident niche and (a) cultured in an *in vitro* niche, prior to transplantation into the infarcted heart, or (b) the local microenvironment can be modulated in order to recruit CPCs to the injured area. The aim of both approaches is to regenerate the myocardium thanks to CPC proliferation and differentiation into cardiomyocytes.

For cell therapy, cells are isolated from their “resident niche” and expanded in an “*in vitro* niche,” prior to transplantation into the “diseased niche” of the infarcted heart tissue (Figure 2(a)). An alternative to cell therapy is to promote the regeneration provided by endogenous CPCs, for instance by promoting the migration of CPCs to the damaged cardiac area (and to the “diseased niche”), as well as their proliferation and differentiation (Figure 2(b)). Another potential approach is to generate new, or engineered, microenvironments for the cells, in order to recreate optimal conditions to enhance their regenerative potential. Currently, there is a lack of knowledge on the composition and similarities of these three niches and on the interplay between CPCs and the niche components.

Therefore, in this review, we highlight the key elements of all potential CPC niches and discuss the interplay between CPCs and the niche components. Improved knowledge on the CPC niches and the CPC-niche interactions will enhance our insight into CPC behavior and the influence of the niche on CPC regenerative capacity, which can ultimately help modulate the microenvironment to promote the regenerative potential of CPCs. In the last part of this review, we therefore provide an overview on recent advances in the field of engineered cardiac microenvironments, which can represent the next step in exploring and modulating the CPC niche and CPC behavior for cardiac repair.

## 2. The Cardiac Resident Progenitor Cell

The presence of CPCs in the fetal and adult heart in mammals (including humans) has been extensively described (reviewed by [11]). Yet, CPCs are not conclusively defined and the nomenclature stem/progenitor cell is often used in a generic sense. However, whereas “stem cells” replicate indefinitely and are pluripotent, “progenitor cells” can only divide a limited amount of times and are multipotent. To prevent misunderstanding, in this review, we propose a definition for CPC, based on key characteristics and functions of these cells. To be identified as a *cardiac progenitor*, a cell should (1) reside throughout the heart in both embryonic and adult stage and be (2) self-renewing and (3) multipotent, that is, able to differentiate in minimally three of the four cardiac cell types (cardiomyocytes, endothelial cells, smooth muscle cells, and fibroblasts). Furthermore, the cell should (4) be activated during cardiac injury and have regenerative potential proven by the fact that (5) transplantation of these cells into the diseased heart has favorable effects on cardiac function.

In this section, we will describe the populations of cells that we include among CPCs, as well as others that are often classified as CPCs but that we exclude from the CPC definition (Table 1), with a focus on their regenerative potential.

### 2.1. Cardiac Side Population Cells

The identification of cardiac progenitor cell population in the heart goes back to the beginning of the twenty-first century. Since 2002, several studies described the presence in the adult mouse heart of cardiac side population cells (cSPCs) [29–32], which were later identified also in fetal and adult hearts of rats and humans [33–35].

*In vitro* studies have proven the ability of these cells to self-renew with retained side population phenotype [14, 30, 32, 36], as well as their multipotency. Differentiation potential into cardiomyocytes [14, 33, 37–40], endothelial cells [36, 39, 41], and smooth muscle cells [39] was confirmed by transplantation studies into the injured heart [31, 33, 41]. Differentiation of side population cells into fibroblasts has so far only been shown after transplantation into the cryoinjured rat heart [33].

cSPCs are activated in murine injured hearts [42, 43], and more clinically relevant, they were also activated in human hearts in response to injury [6, 38, 44]. The regenerative potential of the cardiac side population was tested in three transplantation studies in animal models of cardiac injury. Only one of the performed transplantation studies [30, 31, 33] assessed the functional recovery, reporting increase of injection fraction [30].

### 2.2. c-kit^+^ and Sca-1^+^ Cardiac Cells

While no single marker exclusively identifies CPCs, there is a strong agreement that specific surface markers, like type III tyrosine kinase receptor c-kit (CD117) and Sca-1, identify cardiac progenitor populations. During early development, both markers are primarily haematopoietic stem cell markers present in the bone marrow. In 2003, both cells types were identified in the myocardium of adult rodents [10, 45]. These cell populations are heterogeneous and thereby share similarities but are also distinct, although it is suggested that they both originate from the same resident precursor cell [46].

#### 2.2.1. c-kit^+^ Cardiac Cells

c-kit^+^ cardiac resident progenitor cells are probably the most studied CPC population. Following the discovery in 2003, the presence of c-kit^+^ cells was confirmed not only in human [47] and mouse [48–50] from the developing to adult heart [51–54] but also in other mammals, including dog [55], pig [56], and sheep [57].

Self-renewal of c-kit^+^ CPCs has been assessed *in vitro* [45, 47, 55], and c-kit^+^ cells appear to be the most undifferentiated progenitor population [58]. Despite controversy about the multipotency of c-kit^+^ CPCs [46], several *in vitro* approaches revealed differentiation potential towards all four cardiac cell types, although the extent of differentiation is species- and developmental stage-dependent [10, 35, 47, 50, 55, 59, 60]. In fact, transplantation studies showed that c-kit^+^ CPCs are more prone to differentiate toward endothelial and smooth muscle cells rather than cardiomyocytes and fibroblasts [8, 45, 50, 61]. This suggests that fetal and neonatal-derived c-kit^+^ CPCs only have potential to differentiate into cardiomyocytes, while adult-derived cells are more predisposed to differentiate into vascular cells only [48, 62].

A number of studies have verified the presence of c-kit^+^ cells in hypertrophic adult human hearts [12], after myocardial infarction [6, 50, 53, 63] and after ischemia/reperfusion injury [64, 65]. In chronic heart failure, both increases and decreases in cell numbers have been described [6, 66, 67].

Transplantation of adult c-kit^+^ cells in preclinical studies in rodents revealed that attenuation of scar formation and left ventricular function [47, 68–70] was mainly induced by a paracrine mechanism. Although more preclinical research is needed to fully understand the contribution of c-kit^+^ cells to cardiac regeneration, the first clinical trial of Cardiac Stem Cell Infusion in Patients with Ischemic Cardiomyopathy (SCIPIO, NCT00474461) was conducted [71, 72]. The data of this randomized phase 1 trial reported the induction of myocardial regeneration by c-kit^+^ cells [72–74]. Despite the limited number of patients and the lack of placebo controls in this randomized phase 1 trial, as well as the expression of concern by *The Lancet*, both preclinical and clinical outcomes suggest a contribution of c-kit^+^ cells to cardiac repair.

#### 2.2.2. Sca-1^+^ Cardiac Cells

Resident Sca-1^+^ cells are found in fetal and adult mouse and human hearts, in the atria, the intra-atrial septum, the myocardium, and the epicardium [10, 12, 54, 73, 74].

Human Sca1^+^ cells harbor telomerase activity, which characterizes their proliferative potential and their ability to self-renew [73, 75, 76]. They display a mesenchymal profile and have gene expression comparable with c-kit^+^ cells, although murine Sca-1^+^ CPCs have shown the highest correlation with cardiomyocytes and thereby seem to be the most committed to cardiomyogenic differentiation [58]. Culture-expanded Sca-1^+^ cells can be differentiated into cardiomyocytes in the presence of oxytocin or 5-azacytidine treatment [13, 75, 76], and the cardiac differentiation potential is enhanced by addition of transforming growth factor-beta (TGF-*β*) [75, 76]. Next to cardiomyocytes, Sca-1^+^ cells can differentiate into endothelial cells and smooth muscle cells, as observed both *in vitro* [10, 74, 75, 77] and *in vivo*. Transplantation of isolated adult murine Sca-1^+^ induced revascularization and revealed differentiation into cardiomyocytes and endothelial cells in infarcted mouse heart [13, 74, 76]. Similarly to c-kit^+^ cells, versatility of differentiation of Sca1^+^ cells is developmental stage- and subpopulation-dependent. Whereas fetal cells are very suitable for cardiomyogenic and angiogenic development, adult cells prefer smooth muscle cell differentiation [78].

As for cSPCs and c-kit^+^ cells, Sca-1^+^ cells are present in the hypertrophic human heart [12] and the number of resident cells is expanding after myocardial infarction [74]. Transplantation of both fetal and adult Sca-1^+^ cells, of both murine or human origin, into the mouse injured heart limits structural and functional deterioration and thereby attenuates impairment of contractility. This regenerative potential is mediated by differentiation of Sca-1^+^ cells and via paracrine mechanisms [74, 76, 79, 80].

Despite these functional benefits of Sca-1^+^ cells *in vivo*, no clinical trials are conducted. The fact that there is no Sca-1 homologue present in the human genome raises questions about the epitope on human Sca-1^+^ CPCs that is recognized, which hampers their clinical application [81]. Interestingly, a panel of antibodies has recently been published that were specifically raised against resident human Sca-1^+^ CPCs. These antibodies, such as mAb C19, recognize CPCs in human heart tissue, and isolated C19^+^ cells have CPC characteristics and differentiate into the same lineages as Sca-1^+^ CPCs. This discovery might be a step forward for the application of these human CPCs in clinical trials [82].

### 2.3. Isl1^+^ Cell and Cardiospheres

Other cardiac cell types are often included among putative CPCs, such as Isl1^+^ cells and cardiospheres [46, 60, 83–86]. These cells reside in the heart; however, they do not (yet) respond to our definition of CPCs. Hereafter, we report what is known about Isl1^+^ cells and cardiospheres and explain why we excluded them from the CPC classification.

#### 2.3.1. Isl1^+^ Progenitors

LIM-homeodomain transcription factor Isl1 positive cardiac cells share many of the characteristics of the CPCs described so far. Isl1^+^ cells are present in the developing heart [87], and the number of Isl1^+^ cells residing in the heart is substantially decreasing from fetal to neonatal and adult stages [62, 87–91]. Their distribution is comparable with Sca-1^+^ cells; the location is conserved between rodents and human [90]. Postnatal Isl1^+^ cells can proliferate on cardiac mesenchymal feeders [85, 92, 93], and they have been shown to differentiate into cardiomyocytes, endothelial cells, and smooth muscle cells [85, 90, 94]. Recently, local upregulation of Isl1^+^ after ischemia/reperfusion in the adult mouse heart has been observed [95]. However, at the moment, there is no evidence of the regenerative potential of these cells, due to a lack of data regarding the beneficial effects on cardiac function after transplantation into the diseased heart. Nevertheless, there is interest in their therapeutic value, as shown by a clinical trial designed by Assistance Publique—Hôpitaux de Paris (NCT02057900).

#### 2.3.2. Cardiospheres and Cardiosphere-Derived Cells

Often included also among CPCs are cardiospheres and cardiosphere-derived cells, first described by Messina et al. in 2004 [96]. Cardiospheres can be obtained from human atrial or ventricular biopsies from embryonic, fetal, and postnatal mouse hearts [96–98]. Cells migrating from the tissue explants spontaneously form cardiac multicellular spheroids when cultured on poly-D-lysine-coated culture plates. The cell monolayer growing after seeding cardiospheres on fibronectin-coated culture plates is known as cardiosphere-derived cells [99]. Although they unquestionably reside in the heart, due to the methods used to obtain cardiospheres and cardiosphere-derived cells, the origin of these cells is difficult to determine. Although most cardiosphere-derived cells in culture are known to express the endoglin marker CD105 [98], this mesenchymal and hematopoietic marker is not exclusive and thus cannot be used to specifically define these cells *in vivo* as CPCs. Moreover, activation after injury cannot be proven due to the lack of a specific marker for these cells. Nevertheless, cardiospheres and cardiosphere-derived cells are self-renewing and can form cardiomyocytes, endothelial cells, and smooth muscle cells [96, 98–100].

Cardiospheres contain a mixed cell population, including c-kit^+^ cells as well as endothelial precursors and mesenchymal cells [99]. Moreover, the expression of ECM proteins and integrins, as well as the gradients of oxygen and nutrients that are thought to occur between the periphery and the core of the spheroid, makes cardiospheres an *in vitro* model mimicking the CPC niche [99, 100]. As such, cardiospheres can be used to study the CPC-niche interactions *in vitro*, beside their potential therapeutic application.

Adult cardiospheres and cardiosphere-derived cells have proven to have beneficial effects on cardiac function in murine and porcine animal models [101–107], and these broadly positive findings have led to two clinical trials, CADUCEUS and ALLSTAR. The Cardiosphere-Derived Autologous Stem Cells to Reverse Ventricular Dysfunction (CADUCEUS, NCT00893360) trial is a phase I randomized study in which patients received cells three months after myocardial infarction [108]. Although the study was not powered, transplantation of cells was safe and led to reduction in infarct size and increase in area of viable myocardium, unfortunately without change in ejection fraction of the left ventricle [108, 109]. The positive outcomes initiated the start of a follow-up phase II clinical trial—Allogenic Heart Stem Cells to Achieve Myocardial Regeneration (ALLSTAR) [110].

Above, we described the various populations of resident CPCs that have been identified in the heart. All these cells have a heterogeneous nature and although they express different markers, they might be more similar than they are different. It is even suggested that all these described CPCs represent the same population and that the difference lies in the method of determination or their differentiation stage [47]. It is therefore important to precisely characterize and purify the CPC population, and further research is required. Nonetheless, although CPCs might not be rigorously defined, these cells have potential in cardiac regeneration.

## 3. The CPC Microenvironment

The regenerative potential of the heart is determined not only by the characteristics of CPCs but also by the influence of the microenvironment on their functions. In this section, the key components of the CPC niche will be described. These are (1) cellular components, represented by supporting cells; (2) cyclic strain, as provided by the cardiac beating; (3) extracellular matrix, which provides both mechanical and biochemical stimuli; and (4) soluble factors (such as cytokines) and oxygen tension, which can play a pivotal role in determining stem cell behavior (schematically represented in Figure 1(a)).

### 3.1. Supporting Cells

**Figure 3:**
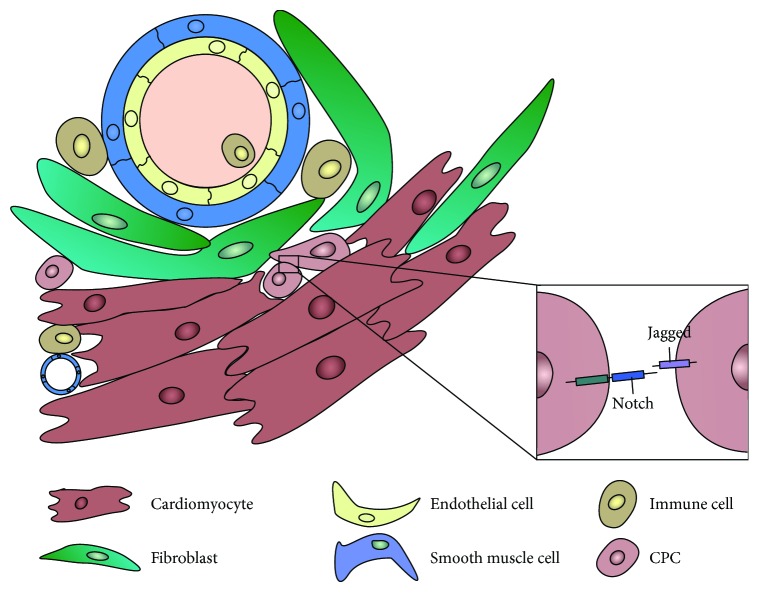
Cell-cell interactions in the CPC niche. CPCs interact with each other and with supporting cells (cardiomyocytes, fibroblasts, endothelial cells, smooth muscle cells, and immune cells), both via direct cell-cell signaling (such as the Notch pathway) and paracrine signaling.

In both healthy and diseased hearts, cells interact with each other directly via cell-cell contact or indirectly by the expression of paracrine factors (Figure 3). Interactions can be isotypic (same cell types) or heterotypic (cells of different phenotypes) and the crosstalk between different populations will affect not only the cardiac function but also the regenerative potential. These interaction processes are complex and mostly unknown in the CPC niche. The role of the direct contact between CPCs and supporting cells is difficult to unravel. Most knowledge is derived from *in vitro* experiments, and crosstalk outcomes are mainly based on paracrine effects. In this section, we will focus on the interactions of CPCs with supporting cells and their importance for cardiac repair.

#### 3.1.1. Cardiomyocytes

In the first *in vivo* study about the cardiac niche and putative supporting cells, connexins and cadherins were detected in cellular contacts between CPCs and cardiomyocytes as well as between CPCs and fibroblasts [27, 47, 111]. However, these connections were not observed between CPCs and endothelial cells and between CPCs and smooth muscle cells [27]. Cardiomyocytes are able to transfer information to CPCs (and vice versa) through gap junctions. Coculture of CPCs with cardiomyocytes promotes their expansion and results in beating CPCs, together with expression of cardiomyocyte-specific proteins and well-organized sarcomeres [14, 37, 90, 96, 112], a process probably regulated by TGF-*β* [113, 114] and indirectly via the Wnt/beta-catenin signaling system [115]. Therefore, coupling of CPCs with cardiomyocytes is critical to control the cardiac fate, and lack of appropriate interaction may hamper CPC differentiation [116]. However, cardiomyocytes might not solely stimulate differentiation toward the cardiomyogenic lineage. In fact, under hypoxia, cardiomyocytes produce vascular endothelial growth factors (VEGF), which might induce endothelial differentiation of the CPCs [115]. At the same time, CPCs can express growth factors and cytokines, which besides being necessary for their proliferation and senescence [117] are also important for cardiomyocyte proliferation, cell survival, and prevention of hypertrophy [118].

#### 3.1.2. Endothelial Cells

Since CPCs are often found in the perivascular area, interaction with endothelial cells and smooth muscle cells is plausible, although cell-cell interactions were not observed [27]. It can be hypothesized that endothelial cell-CPC interaction is regulated via Notch, since Notch receptors are predominantly expressed by the vascular endothelium [119]. Notch signaling is crucial for cell fate decisions that underlie cardiomyogenic and vessel formation [119, 120]. Since Notch signaling is a highly conserved pathway that acts via cell-cell contact, it will be discussed in more detail later on. Indirect interactions between endothelial cells and CPCs, via the production of VEGF, might not only promote CPC migration [121] but also regulate CPC differentiation towards endothelial or smooth muscle cells [39, 41].

#### 3.1.3. Immune Cells

Myocardial injury causes inflammation by activation of immune cells, which are involved in cardiac repair as well as scar tissue formation. Hence, crosstalk between CPCs and immune cells is likely to take place, although there are no proven interactions. Transplantation studies revealed that CPCs are able to dampen the immune response and thereby influence cardiac repair [122]. However, the mechanisms underlying CPC modulation of the immune system are not completely unrevealed, as it is the case for mesenchymal stem cells [123–126].

*(1) Macrophages*. Macrophages are a heterogeneous population of both protective and cytotoxic cells. They play a cardioprotective role by maintaining cardiac homeostasis via interactions with other cardiac cells [127]. Macrophages are able to produce growth factors (e.g., IGF-1, VEGF, and TGF-*β*), which stimulates CPC proliferation and induces differentiation towards both cardiomyocytes and endothelial cells [128, 129]. On the other hand, CPCs are able to polarize macrophages away from their proinflammatory phenotype, although the exact mechanism behind it is unclear. It is not known toward which cell type the polarization acts, although it was proven not to be toward the anti-inflammatory phenotype [130]. We therefore assume that a cardioprotective effect arises from the interaction between macrophages and CPCs.

*(2) Natural Killer Cells*. Natural killer cells, a subset of the innate lymphoid cell compartment, are effectors of the innate immune system, which are essential in allogeneic transplantation. Their cytotoxic effects are mediated by exocytosis of granules that perforate the target cell to trigger apoptosis [131]. Little is known about these cells and CPCs, but a recent study by Boukouaci et al. revealed that CPCs are protected from killer cell cytotoxicity within an inflammatory context [132]. On the other hand, CPCs are able to downregulate the toxicity of natural killer cells and bias cytokine secretion towards an anti-inflammatory state. Retention of CPCs is improved by this crosstalk with natural killer cells and contributes to cardiac regeneration [132].

*(3) Mast Cells*. Mast cells are bone marrow-derived precursors, and although their number increases in the failing heart, their exact role in cardiac disease and regeneration is understudied. Moreover, mast cells express similar markers as CPCs [133]. It is known that CPCs share distinct characteristics with mast cells, but not all CPCs are mast cells [133]. Both mast cells and CPCs are located in the perivascular area, although cell contact is not reported. Paracrine effects can be assumed since mast cells produce several cytokines, growth factors, and angiogenic factors that are all involved in cardiac repair [134]. During mast cell degranulation, TGF-*β* is released, which as described earlier is important for CPC differentiation [135].

#### 3.1.4. Stromal Cells

*(1) Fibroblasts*. Together with cardiomyocytes, fibroblasts were the first supporting cells of CPCs to be discovered [27, 47]. Like cardiomyocytes, fibroblasts are connected to CPCs via gap and adherens junctions. Not only do fibroblasts maintain the supporting matrix of the CPC niche [115] (the importance of the ECM-cell interaction will be discussed later on in a dedicated paragraph), but they also might influence the differentiation potential of CPCs. It has recently been shown that fibroblast-conditioned medium can induce differentiation via the Wnt signaling pathway [136] and that fibroblasts produce angiogenic and antiangiogenic factors [115]. Fibroblasts originate mainly from the epicardium [137]; therefore, interactions between epicardium-derived cells (EPDCs) and CPCs need to be described.

*(2) Epicardium-Derived Cells (EPDCs)*. CPCs are often found in the subepicardial region, which mostly consists of EPDCs. EPDCs have a crucial modulatory role during cardiac development, and their activation after injury [138–141] also suggests the same role in the adult heart [142]. Due to these characteristics, some groups even suggest that the epicardium is a source of progenitor cells [143, 144]. The presence of CPCs near the epicardium and EPDCs suggests that important interactions occur between CPCs and EPDCs. Previous research showed that EPDCs stimulate the migration and proliferation of CPCs [141, 145, 146]. Coculture of CPCs with EPDCs revealed induction of metalloproteinases and their inhibitors, which affected infarct size [146]. In fact, matrix remodeling is not only important to prevent cardiac dilation after injury, but it also plays a role in maintaining the supporting network of the CPC niche. Moreover, coculturing stimulated angiogenesis and thereby improved cardiac function. The interaction between CPCs and EPDCs is reciprocal and results in synergistic action, leading to improved cardiac function. This beneficial effect is at least partly explained by paracrine stimulation [146].

*(3) Telocytes*. Another stromal cell type which is present in the subepicardial region is the telocyte, formally known as interstitial Cajal-like cells [147]. Telocytes are in close vicinity with CPCs, and stromal synapses and adherens junctions are formed between the cells both *in vitro* and *in vivo* [148, 149]. These adherens junctions not only control the retention of CPCs but also might be important for division and migration of CPCs [149]. Therefore, it is assumed that telocytes provide guidance and nursing for CPCs to stimulate their activation, proliferation, and differentiation leading to cardiac repair [148, 150, 151]. Furthermore, telocytes produce growth factors (e.g., VEGF) [152] and macromolecular signals, such as microRNAs [153], which might influence the differentiation potential of CPCs [151].

#### 3.1.5. Cell-Cell Signaling via Notch in CPCs

Cell-cell contact-dependent signaling is an essential component of the niche and has an important influence on cellular behavior. Notch signaling is a fundamental and highly conserved pathway that acts via direct cell-cell communication and has a key role in the heart. Notch regulates a number of cell functions, such as survival, proliferation, and differentiation, as well as tissue development and homeostasis. In mammals, four Notch proteins have been identified (Notch1–4), which can bind to ligands of the Delta or Serrate/Jagged families expressed by neighboring cells, as extensively reviewed elsewhere [154, 155] (Figure 3). Following cleavage by ɣ-secretase, Notch intracellular domain (NICD) is released and translocates to the nucleus, where it regulates the expression of target genes, such as members of the Hes and Hey families [154–156], as well as Nkx2.5 in cardiac cells [157].

Notch signaling represents an essential element of the cardiac microenvironment. Firstly, Notch plays a crucial role in cardiomyogenesis, and Notch mutations have been linked to several congenital heart and heart valve defects [154–156]. Active Notch signaling is needed for CPC differentiation [158], whereas in cardiomyocytes, Notch is activated during embryonic development [155, 159] and inactivated during maturation [160] and after birth (reviewed by [159]). Secondly, several studies demonstrated the reactivation of Notch in adult cardiomyocytes after MI, in small animal models [157] as well as in humans [161, 162]. This shows the key role of Notch in cardiomyocyte survival [163, 164] and cardiac repair after injury [159, 165].

In the adult mouse, about 60% of c-kit^+^ CPCs expresses the Notch1 receptor, and signaling with surrounding cells, either CPCs or myocytes, is mediated by Jagged1 [157]. Notch signaling strongly depends on timing and dosage [166, 167]; it is needed for proliferation and expansion of the CPC pool [160] and is essential for CPC cardiomyogenic differentiation [157, 158]. The activation of Notch1 by Jagged1 in mouse c-kit^+^ CPCs promotes the nuclear translocation of N1ICD and enhances its colocalization with the cardiac transcription factor and Notch target gene Nkx2.5 [157]. However, Notch becomes undetectable when differentiating CPCs lose their proliferative capacity, showing that its downregulation is needed for terminal differentiation [160]. Moreover, overexpression of Notch1 in mouse c-kit^+^ CPCs leads to improved resistance to oxidative stress, and injection of these cells in mouse infarcted hearts resulted in an enhanced cardioprotective effect, as shown by smaller infarct length and area, functional improvement, and larger capillary density as compared to control cells (where endogenous Notch was activated via Jagged1) [120].

In view of these findings, Notch has been proposed as a potential therapeutic target for treating myocardial disease. Hydrogels functionalized with a peptide mimic of Jagged1 were shown to activate Notch signaling in rat c-kit^+^ CPCs, and injection of CPCs embedded in these hydrogels led to improved cardiac repair in a mouse MI model [168].

Yet, very little is known about Notch in human CPCs. Recent studies, by our group and by others, have studied Notch signaling in cardiac progenitors cultured as multicellular spheroids, or cardiospheres (if isolated from adult myocardial tissue). The spheroid model better mimics the *in vivo* cell-cell interactions as compared to cell monolayers and therefore represents an interesting and promising model to study cell-cell signaling (see paragraph 4, “Approaches to modulating the CPC microenvironment”; [169]). The formation of cardiospheres from adult explant-derived cells increased the expression of Notch1 and Notch3 receptors [170]. Moreover, multicellular spheroid formation was shown to activate Notch signaling in both fetal and adult CPCs and more evidently so in combination with hypoxic culture, indicating the pivotal role of niche-like environmental conditions on a fundamental cellular pathway such as Notch signaling.

Understanding the role and activity of this key regulatory pathway in human endogenous CPCs will be crucial for the improvement of CPC-based cardiac regeneration therapies.

### 3.2. Extracellular Matrix

Cardiac cells are surrounded by a highly organized and dynamic network, known as the ECM that forms the cardiac tissue [171]. The cardiac ECM is composed of different proteins, proteoglycans, and glycosaminoglycans that form a fibrillar mechanical support in which cells are embedded. These structural components include collagen types I, III, and V, as well as elastin that provides resilience to the cardiac tissue [172–174]. Furthermore, proteoglycans such as tenascin-C and decorin contribute to the cardiac tissue and are crucial for the stability and integrity of the ECM [175, 176]. Next to structural components, the ECM is composed of nonstructural elements that regulate important cellular functions, such as adhesion, proliferation, and differentiation. These are primarily type IV collagen, laminins, and fibronectin [172, 177]. Moreover, within the ECM network, different cell types secrete soluble macromolecules in the extracellular space, such as VEGF, TGF-*β*, and stromal cell-derived factor 1-*α* (SDF1-*α*), which regulate and stimulate important cellular processes [178–180].

Cardiac fibroblasts are primarily responsible for the production and remodeling of ECM [181], both under healthy and under pathological conditions. During and following a myocardial infarction, fibroblasts become activated and secrete an abundance of ECM components to compensate for the loss of cardiomyocytes. Eventually, this leads to excessive ECM formation and scarring, with adverse effects on the contractility of the cardiac tissue. Altogether, this fibrotic environment might not be ideal for the injected progenitor cells. For this reason, the effect of the ECM environment on progenitor cell survival and function should be investigated to improve the CPC contribution to cardiac regeneration. Below, recent literature on the interaction of CPCs with native and/or synthetic ECM *in vitro* and *in vivo* is summarized and discussed, with a focus on ECM properties such as stiffness, architecture, and composition.

#### 3.2.1. Integrins

CPC adhesion to its environment is essential for the connection between intracellular components and the ECM. Via focal adhesions (FAs), CPCs can sense their environment and respond accordingly. In general, FAs are transmembrane protein complexes that directly link ECM components or other cells to intracellular actin junctions, intermediate filaments, and sarcomeres [182]. Important components of these transmembrane proteins are integrins, which are heterodimers consisting of a combination of *α* and *β* subunits. In mammals, 24 types of receptors can be formed and each combination has a specific binding affinity to a different ECM component. For CMs, the most occurring integrins are *α*1*β*1, *α*5*β*1, and *α*7*β*1, which bind specifically to collagen type I (COL), fibronectin (FN), and laminin (LN), respectively, with *β*1 being the prevalent *β* subunit [182]. Furthermore, the protein expression of different types of integrin also changes from neonatal CMs, where the dominant subunit is *α*5, to adult CMs, where the *α*5 is replaced by *α*7 subunit [182, 183]. Similar changes in integrins expression can also be observed in response to pathological conditions [182]. Human fetal CPCs subjected to cardiomyogenic differentiation protocol *in vitro* showed unvaried expression but increased clustering of integrin *β*1, indicating FA maturation and improved mechanosensing with early cardiac differentiation [184]. These studies underline the importance of integrins in the heart and suggest that the FA expression of CPCs and interactions with specific ECM components should also be studied to be able to guide CPCs towards specific lineages.

#### 3.2.2. CPC-ECM Interactions

*(1) ECM Composition*. The versatility of interactions between CPCs and their direct surroundings generates the possibility to obtain highly regulated and regenerative cellular responses via intracellular signaling. It is currently known that the stiffness, composition, and/or structure of the natural ECM has an effect on progenitor commitment *in vitro* [185]. More importantly, the response that the ECM evokes on cells are different depending on the cell type. This has been tested by culturing cardiac progenitor cells on different substrates *in vitro* and studying the cellular behavior and functions associated with cardiac regeneration. The first studies were initiated by French et al., who studied c-kit^+^ Sprague-Dawley rat CPC behavior, that is, cardiomyogenic gene expression, cell survival, and proliferation, cultured on decellularized porcine ventricular ECM (cECM) or standard collagen type I (COL), which more closely resembles the biochemical composition of a scar following a MI [186]. Interestingly, early cardiac genes for GATA-binding protein-4 (GATA-4), Nkx2.5, *α*-myosin heavy chain, and troponin C and T were increased when CPCs were cultured for 2 days on cECM compared to COL. Moreover, fibroblast and endothelial/smooth muscle cell-specific genes decreased and remained constant, respectively, for CPCs cultured on cECM compared to COL. In a recent study, human Sca-1^+^ cells originating from either fetal heart (fCPCs) or adult hearts (aCPCs) were encapsulated and cultured in three-dimensional (3D) hydrogels consisting of either cECM or COL [187]. Similarly, gene expression of early cardiac markers, that is, GATA-4, Nkx2.5, myocyte enhancer factor 2c (Mef2c), and myosin light chain 2v (MLC2v), increased when fCPCs and aCPCs were cultured in cECM compared to COL after 4 days. Furthermore, after 7 days, these markers increased when fCPCs were cultured in cECM and remained constant for aCPCs. One explanation for the minimal increase in cardiac markers after a longer period of time could be the development of an endogenous microenvironment by CPCs that decreases the early biochemical effect of cECM. Additionally, improved proliferation and survival was observed for both cECM-coated surfaces and for CPCs encapsulated in cECM hydrogels.

These findings indicate the importance of the biochemical composition for the early maturation of fCPCs towards cardiac specific lineages. Nevertheless, more information is needed on the specific ECM composition to be able to understand which of the components generates the beneficial response of CPCs towards cardiac-derived ECM, considering the fact that CPCs reside in a specific, yet complex, niche that strongly determines their behavior [27]. In a follow-up study by French et al., CPCs were cultured on cECM and were COL-, FN-, and LN-functionalized. In a follow-up study by French et al., CPCs were cultured and subjected to different cyclic strains on Bioflex plates functionalized with cECM, COL, FN, or LN [188]. Proliferating cell nuclear antigen (PCNA) was used as a measure to determine the proliferation response of CPCs cultured for 30 hours on the different substrates. The highest number of PCNA positive cells was observed on substrates functionalized with fibronectin, demonstrating the benefit of a single ECM component compared to the whole complex cardiac ECM. Fibronectin has been shown to be crucial for the expansion of human CPCs during development and after a MI [189]. Additionally, endogenous fibronectin production by human CPCs has been observed after 7 days in static culture [15]. Interestingly, the beneficial effect of fibronectin on the proliferation of CPCs seems to diminish at strain magnitudes of 10–15%. These findings suggest the importance of fibronectin on the initial proliferation response of CPCs; however, the effect can be overruled by other microenvironmental components such as cyclic strain and/or stiffness.

*(2) ECM Stiffness*. The data described so far suggest that an ideal biochemical environment is not sufficient to completely obtain the desired regenerative response, but that mechanical and/or structural stimuli also contribute to a favorable response. To illustrate this, c-kit^+^ human pediatric CPCs were cultured in neonatal or adult ECM derived from Sprague-Dawley rats and combined with fibrin to create 3D hybrid hydrogels with a range of Young's moduli, that is, 2, 8, 14, and 32 kPa [190]. By increasing Young's modulus of the neonatal and adult ECM-fibrin hybrid hydrogel from 2 to 8 kPa, the gene expression of cardiac titin decreased, whereas it increased at higher moduli. These findings suggest that ECM stiffness has an effect on the genetic behavior of CPCs in terms of cardiac titin expression. Encapsulating CPCs in an environment with a stiffness that resembles the native mechanical properties would provide better conditions to study the development of CPCs into mature cardiomyocytes. However, this is complicated by the fact that stiffness values may differ between neonatal, fetal, and adult heart, between healthy and diseased conditions, and also between species [191].

To study the effect of ECM stiffness on cardiac stem/progenitor cell maturation, hydrogels with time-dependent and development-mimicking stiffnesses were developed based on thiolated-hyaluronic acid (HA) and crosslinked with poly(ethylene glycol) (PEG) diacrylate. By growing precardiac embryonic stem cells on HA-hydrogels with elastic moduli ranging from 1 to 10 kPa, a 60% increase in myofibril orientation and a 3-fold increase in Troponin T expression was observed compared to cells grown on mechanically static polyacrylamide hydrogels [192]. A recent example of modulating the ECM stiffness is shown by Choi et al., where a sol-to-gel transitional gelatin-PEG-tyramine (GPT) hydrogel with tunable mechanical properties was developed [193]. Hydrogels with elastic moduli of 1.8, 2.8, 5.8, and 8.1 kPa were created by varying the H_2_O_2_ concentration. Interestingly, CPCs isolated from 9-week-old Sprague-Dawley rats showed inhibited f-Actin organization and decreased proliferation in stiffer GPT hydrogels (elastic moduli of 5.8 and 8.1 kPa) compared to lower stiffnesses (1.8 and 2.8 kPa) [197]. However, an enhanced expression level of early cardiac differentiation markers was observed in GPT hydrogels with higher elastic moduli. These results strongly suggest an inhibition of proliferation and enhancement and differentiation in cardiac stem/progenitor cells as a result of increasing the ECM stiffness.

### 3.3. Cyclic Strain

The adult human heart beats 60–100 times per minute every day. Cells that reside in the myocardium are constantly subjected to this mechanical loading, which thus represents a significant component of the cardiac microenvironment that can influence the regenerative response of resident CPCs. However, while many studies have focused on the mechanoresponse of contractile cardiomyocytes, the effect of cyclic strain on CPCs has only been investigated by a few research groups. We recently elucidated the mechanoresponse of human Sca-1^+^ CPCs. Cells were cultured on 2D substrates coated with collagen IV, which together with laminin represents the main component of the cardiomyocyte basement membrane. Whereas undifferentiated CPCs did not show a preferential orientation upon application of uniaxial cyclic strain, CPCs in the early stage of cardiomyogenic differentiation (predifferentiated) oriented perpendicularly to the main direction of the stretch (strain avoidance behavior) after 48 hours [184]. The different responses appear to be due to the development of the mechanosensing structures, such as focal adhesions (FAs) and actin stress fibers (the *mechanosome*), that we demonstrated to occur during the early phase of cardiac differentiation [184]. In the study of French et al. [188] mentioned above, cyclically strained rat c-kit^+^ CPCs displayed a different orientation response when cultured on different ECM coatings. After 24 hours, rat CPCs displayed a strong strain avoidance response on fibronectin and collagen I. On the other hand, the strain avoidance response on cECM was much weaker as compared to collagen I and fibronectin, whereas almost no strain avoidance was observed on laminin [188]. Taken together, the reported studies suggest that CPCs on the natural cardiac ECM are less responsive to cyclic strain as compared to single ECM components (fibronectin, collagen I). Furthermore, the mechanoresponse of CPCs is weakened on certain ECM proteins (laminin, collagen IV). It is tempting to speculate that this behavior might be related to the affinity of different integrins for the ECM proteins. It would be interesting to investigate which integrins are expressed by the CPCs on the different substrates, and especially on the naturally derived cardiac ECM, and relate this expression pattern to the CPC mechanoresponse (as previously done in other cell types by [194, 195]).

It should be noted that the above studies are limited by their 2D setup, which does not resemble the 3D physiologic environment. In a study by van Marion et al. [196], the effects of human Sca-1^+^ CPC engraftment in collagen I/Matrigel hydrogels were investigated. Whereas CPCs showed a random orientation in stress-free hydrogels, in statically constrained hydrogels, they aligned along the direction of the strain after 24 hours. This effect was even more pronounced at day 9 of culture, showing that CPCs become readily mechanosensitive in 3D. Furthermore, already after 24 hours of culturing in the 3D hydrogels, the cardiac differentiation markers were upregulated as compared to the 2D culture, indicating an increased differentiation capacity of the CPCs towards the cardiomyocyte phenotype in 3D.

Detailed investigation of the mechanosensing of (human) CPCs in 3D environments is needed in order to provide a closer clue of the response of these cells to the mechanical stimuli provided by the cardiac microenvironment.

### 3.4. Soluble Factors and Oxygen Tension

After a myocardial infarction, cardiac cells are immediately exposed to hypoxia, due to the temporary lack of oxygen. Hypoxia has been shown to regulate the behavior of several stem and progenitor cells by dramatically influencing fundamental signaling pathways, such as Notch and Oct4, that determine self-renewal and multipotency [197–199]. In response to low oxygen tension, cells express hypoxia-inducible factors (HIFs), with HIF-1*α* being the key mediator of the cellular adaptive response to hypoxia [200]. For example, HIF-1*α* is induced in the ischemic myocardium after MI [201]. HIF-1 directly regulates the transcription of the chemokine stromal cell-derived factor 1 (SDF-1) [202] and its receptors CXCR4 [203], which play an important role in the mobilization of progenitor cells [204–206]. Moreover, the upregulation of SDF-1 in ischemic tissues is directly proportional to the reduction of oxygen tension [202]. The interaction between SDF-1 and CXCR4 plays a crucial role in the mobilization and migration of circulating progenitor cells in ischemic tissues [202, 207, 208].

For cardiac regeneration, the response of resident progenitor cells to low oxygen tension is of great interest, due to the potential contribution of these cells to the cardiac regenerative mechanisms [38]. In this respect, a number of studies on murine CPCs have been conducted.

These cells express the SDF-1 receptors CXCR4 and CXCR7 [209, 210]. In room air conditions (20% O_2_), CPCs show very limited expression of CXCR4; however, under (harsh) hypoxia (0.1% O_2_), expression of both the CXCR4 receptor and the chemokine SDF-1 is greatly enhanced [209]. SDF-1 induces CPC migration in a time- and dose-dependent manner [209, 210]; this SDF-1-induced migration is however abolished by knockdown of CXCR4 or CXCR7 [210], demonstrating the crucial role of the SDF-1/CXCR4 and SDF-1/CXCR7 axis for CPC motility. Pretreatment of murine CPCs with hypoxia results in increased migration toward SDF-1 *in vitro*, suppressed by cell transfection with CXCR4 shRNA [209], once again indicating the key role of the SDF-1/CXCR4 interaction for CPC motility. Additionally, hypoxic pretreatment results in improved recruitment of the CPCs to the ischemic myocardium in a mouse MI model [209], suggesting a potential therapeutic benefit was offered by this procedure.

**Figure 4:**
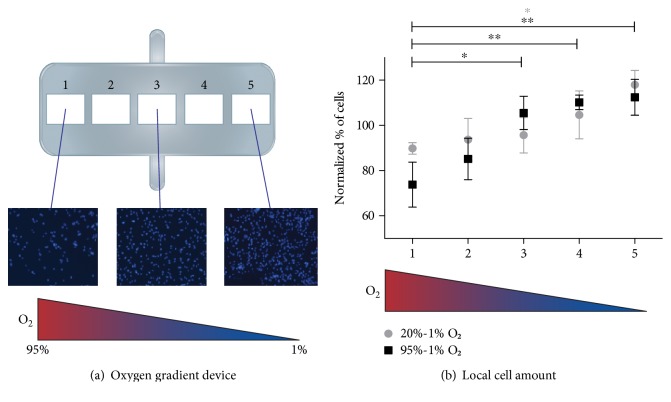
CPCs migrate toward lower oxygen concentration in an oxygen gradient device. (a) In a PDMS device (showed in the schematic representation) where 20% or 95% O_2_ was applied at one end, and 1% O_2_ at the other end, an increasing number of CPCs were observed at the lower oxygen side after 24 hours. Representative images show the increased amount of cells (nuclei stained with Hoechst 33342, blue). (b) The quantification of cell number (normalized to the initial value after seeding) is reported as mean ± SD (*n* = 4; ^∗^*P* < 0.05; ^∗∗^*P* < 0.01).

The response to hypoxia of human CPCs is less known. In a study by van Oorschot et al. [211], human Sca1^+^ CPCs displayed increased proliferation and motility when cultured under low oxygen tension (1% O_2_). The motility and migration of these cells were also enhanced by culture in 1% O_2_-conditioned media [211]. Moreover, human Sca1^+^ CPCs displayed an increase in cell motility directly proportional to the reduction of oxygen tension, similarly to the SDF-1 induction observed in ischemic tissues by Ceradini and Gurtner [212]. In a hypoxia-gradient microfluidics chip, where high O_2_ tension was applied on one end (20% or 95%) and 1% O_2_ at the other end, an increasing number of human Sca1^+^ CPCs was detected after 24 hours towards the condition of the lowest oxygen tension (Figure 4). CPC displayed similar proliferation in all the areas of the chip, thereby suggesting that the higher amount of cells in the hypoxic area is indeed due to CPC migration.

This suggests that, under hypoxic conditions, not only do human CPCs show improved motility but they also release chemoattractants and their receptors. However, the induction of SDF1 and CXCR4/CXCR7 in human CPCs has not been investigated yet. Given the data here reported on murine CPCs and on other human progenitor cells, a mechanism similar to the SDF-1/CXCR4 axis might take place.

## 4. Approaches to Modulate the CPC Microenvironment

**Figure 5:**
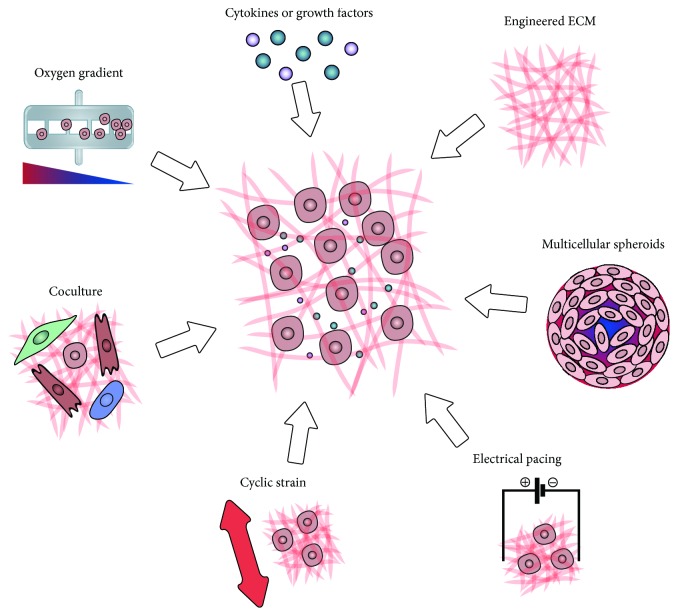
*In vitro* approaches to modulate the CPC niche. Strategies to optimize the regenerative potential of CPCs by modulating their microenvironment include (clockwise) the following: engineering the ECM with synthetic or naturally derived polymers with the right composition and physical properties; recreating a niche-like environment by growing cells as multicellular spheroids; applying electrical pacing and/or cyclic strain; co- and multiculture of different cell types to optimize cell-cell interactions, with or without surrounding ECM; and modulating the cell recruiting potential held by gradients of oxygen and cytokines and growth factors.

The CPC niche is complex and its importance for cardiac differentiation, maturation, and contribution to repair is largely unknown. For a better understanding, engineering approaches to recapitulate the native cardiac microenvironment are required. For recreating the CPC niche *in vitro*, several key components are of importance. In this part of the review, we will focus on current *in vitro* engineering approaches to mimic the cell natural environment (Figure 5).

As previously stated, the CPC microenvironment or niche should display key characteristics, such as optimal biochemical, physical, and mechanical properties, to enhance the regenerative response of CPCs. This ideal microenvironment should therefore stimulate either proliferation or differentiation, or elicit a beneficial effect on the paracrine signaling of CPCs. To date, little is known of the exact characteristics of this ideal niche and what is necessary to obtain optimal CPC contribution to cardiac regeneration.

Multicellular spheroids are scaffold-free spherical cell aggregates that mimic in the most simplistic way the conditions of the niche [99, 100, 213]. As compared to 2D cell culture, spheroids provide improved cell-cell and cell-ECM interactions, as well as gradients of soluble factors, such as oxygen and nutrients [213–216]. Therefore, cell spheroids are used as a model to study cell behavior in a 3D environment that better resembles the *in vivo* conditions. At the same time, they could entail major advantages for clinical use over the injection of cells grown as a monolayer, especially in the treatment of cardiac disease (as extensively described by [169, 216]).

However, an engineered microenvironment could provide more specific signals to CPCs, and its characteristics might be tunable to elicit a distinct response. Currently hydrogels, decellularized ECM, and synthetic matrices are used to create 3D cardiac environments that take into account cell-matrix interactions, as described above. However, though these matrices mimic ECM-like features, they not always resemble the mechanical strength of the native tissue [217]. Unfortunately, only a small amount of studies are performed with CPCs in matrices to create an engineered CPC-niche, although recently these are being increasingly explored [97, 188, 196, 218, 219]. Other cell types, which do not match the definition of CPCs of this review, are more prominently used in hydrogels, and the knowledge gained from these studies might be interesting to engineer the CPC niche. Cardiosphere-derived cells in both alginate [220] or biodegradable poly-(N-isopropylacrylamide) hydrogels showed cardiomyogenic differentiation and proliferation [221] and provided functional benefits [222].

However, these approaches lack to take into account some key aspects of the cardiac microenvironment. By making more use of biomaterials that can form well-defined and “smart” microenvironments, more knowledge can be extracted to finally be able to define the ideal CPC niche. Recently, scalable engineered and force-generating human myocardium was produced under well-defined conditions using embryonic stem cells, induced pluripotent stem cell-derived cardiomyocytes, and fibroblasts [223]. Interestingly, extensive evidence for cardiac molecular maturation and functional tissue formation was obtained using RNA sequencing techniques. According to Tiburcy et al., the most important responses that determine the degree of *in vitro* maturation of human cardiomyocytes are artificial electrical pacing [224], mechanical stimulation (uniaxial and cyclic load) [225], and cocultures with fibroblast-like cells [226]. Finally, they were able to develop a model that can be used to screen drugs, study heart repair or model heart disease, and to study the endogenous repair of CPCs. Another method to create well-defined microenvironments is by making use of micro- and nanoscale engineered biological systems on a chip [227]. With these techniques, different niche components, such as mechanical [228–230], electrical [231], or topographical cues [232], can be carefully modulated and stem cell responses can then be studied in more detail. For instance, Morez et al. have shown improved cardiomyocyte differentiation from CPCs using silicone parallel microgrooves (10 *μ*m wide and 3 *μ*m deep) *in vitro* [233].

Further *in vitro* research is needed on the influence of different niche components on the behavior and regenerative potential of CPCs, in order to make the final next step towards the successful endogenous cardiac repair by CPCs.

## 5. Conclusive Remark

In this review, we have defined cardiac resident progenitor cells according to their behavior and characteristics. Although there is ongoing debate and controversy about the presence of CPCs in the heart and their regenerative potential, a considerable amount of evidence shows that these cells exist and reside in the fetal and adult (human) heart in specific niches. We have highlighted the key components of CPC niches and the interplay of CPCs with niche elements.

As reported, the CPC niche is very complex in structure and composition and the relative and combined effects of individual niche elements on CPC function and regenerative potential is, to date, far from clear. Better understanding of the effect of the niche on cell behavior could lead to strategies to optimize their contribution to cardiac repair. Therefore, we concluded this review by describing how engineering *in vitro* approaches, that take into account the key factors and attempt to mimic the native niche, can enhance the regenerative response of CPCs.

## Figures and Tables

**Table 1:** Summary of the cardiac cell populations described in this review.

Cell type	Cardiac resident		Self-renewal	Multipotent	Activation after injury	Improvement of cardiac function	Defined CPC	[Refs]
	Embryonic	Adult						
Side population	+	+	+	+	+	+	Yes	[6, 9, 11, 14, 29–45]
c-kit^+^ cells	+	+	+	+	+	+	Yes	[6, 8–12, 35, 46–74]
Sca-1 cells	+	+	+	+	+	+	Yes	[6, 9–13]
Isl1 progenitors	+	?	+	+	?	?	No	[9, 11, 47, 62, 88–99]
Cardiospheres	+	+	+	+	?	?	No	[9, 11, 47, 62, 87, 88]
